# Adaptive Landscape by Environment Interactions Dictate Evolutionary Dynamics in Models of Drug Resistance

**DOI:** 10.1371/journal.pcbi.1004710

**Published:** 2016-01-25

**Authors:** C. Brandon Ogbunugafor, C. Scott Wylie, Ibrahim Diakite, Daniel M. Weinreich, Daniel L. Hartl

**Affiliations:** 1 Department of Organismic and Evolutionary Biology, Harvard University, Cambridge, Massachusetts, United States of America; 2 Broad Institute of MIT and Harvard, Cambridge, Massachusetts, United States of America; 3 Department of Ecology and Evolutionary Biology, Brown University, Providence, Rhode Island, United States of America; 4 Department of Global Health and Social Medicine, Harvard Medical School, Boston, Massachusetts, United States of America; University of Washington, UNITED STATES

## Abstract

The adaptive landscape analogy has found practical use in recent years, as many have explored how their understanding can inform therapeutic strategies that subvert the evolution of drug resistance. A major barrier to applications of these concepts is a lack of detail concerning how the environment affects adaptive landscape topography, and consequently, the outcome of drug treatment. Here we combine empirical data, evolutionary theory, and computer simulations towards dissecting adaptive landscape by environment interactions for the evolution of drug resistance in two dimensions—drug concentration and drug type. We do so by studying the resistance mediated by *Plasmodium falciparum* dihydrofolate reductase (DHFR) to two related inhibitors—pyrimethamine and cycloguanil—across a breadth of drug concentrations. We first examine whether the adaptive landscapes for the two drugs are consistent with common definitions of cross-resistance. We then reconstruct all accessible pathways across the landscape, observing how their structure changes with drug environment. We offer a mechanism for non-linearity in the topography of accessible pathways by calculating of the interaction between mutation effects and drug environment, which reveals rampant patterns of epistasis. We then simulate evolution in several different drug environments to observe how these individual mutation effects (and patterns of epistasis) influence paths taken at evolutionary “forks in the road” that dictate adaptive dynamics *in silico*. In doing so, we reveal how classic metrics like the IC_50_ and minimal inhibitory concentration (MIC) are dubious proxies for understanding how evolution will occur across drug environments. We also consider how the findings reveal ambiguities in the cross-resistance concept, as subtle differences in adaptive landscape topography between otherwise equivalent drugs can drive drastically different evolutionary outcomes. Summarizing, we discuss the results with regards to their basic contribution to the study of empirical adaptive landscapes, and in terms of how they inform new models for the evolution of drug resistance.

## Author Summary

The adaptive landscape analogy describes the process of evolution by examining how individual mutations in a gene or genome affect the reproductive success of an organism. In certain cases, it can offer insight into what pathways evolution is likely to take in moving between different phenotypes. The analogy has been used by evolutionary biologists to describe a number of phenomena ranging from how mutations affect hemoglobin function to how bacteria evolve resistance to antibiotics. In this study, we combine computational biology with experimental data to examine how the environment—defined as the type of drugs and their amounts—affects the structure of adaptive landscapes for drug resistance in *Plasmodium falciparum* (the agent responsible for the most deadly form of malaria) with respect to mutations in dihydrofolate reductase (DHFR), an enzyme that plays an important role in drug resistance. We conclude that the environment has a profound effect on how the evolution of drug resistance occurs. In the future, these details should be integrated into models of antimicrobial therapy, as they greatly influence the dynamics of drug resistance evolution.

## Introduction

Evolutionary biology has focused the lens through which we study drug resistance in microbes, helping to create a language to describe the evolutionary relationship between pathogens and therapeutic agents. Simultaneously, drug resistance has become a model problem to explore central concepts in evolutionary theory, including epistasis [1–4], robustness [5] and extinction [6]. In recent years, the adaptive landscape analogy has been applied in various infectious disease contexts [1,7–10], often using combinatorial approaches to identify probable trajectories for the evolution of drug resistance [3,11–16]. Most studies of this kind use the drug concentration that cuts the replication rate in half (IC_50_), the minimum inhibitory concentration (MIC) or related resistance metrics to predict the pathways through which resistance evolves under the assumption that the most resistant variants are preferred in the process of evolution towards maximal drug resistance. This assumption is based on an incomplete appreciation of the growth rate vs. drug concentration curves that generate the IC_50_ and MIC values. Specifically, the IC_50_ and MIC data each intrinsically restricts, in a different way, the environmental dimension over which adaptive landscapes vary, but few studies have examined this area either theoretically [17–19] or empirically [8,15,20].

Further interrogation of the environmental dimension of adaptive landscapes for drug resistance may be useful in the ongoing quest to develop rational strategies to prevent the rise and spread of drug resistance [21–27]. Such inquiry might also be relevant to addressing existing questions regarding how to most effectively treat a malaria infection [25,28–30], and how widespread resistance arises in the first place [31]. As answers to these questions remain elusive, the evolutionary problem of drug resistance can benefit from new models and perspectives.

In this study, we use empirical data and simulations to study the interaction between adaptive landscapes and two environmental dimensions: drug type and concentration. We do so in *Plasmodium falciparum*, the causative agent of the most deadly form of malaria. First, we compare the growth curves for all 16 combinatorial mutants across drug types and concentrations, and ask whether the landscapes for the two drugs display cross-resistance as commonly understood. We then reconstruct all accessible adaptive trajectories for the evolution of drug resistance across drug environments, and observe how their topography changes as a function of environment. Next we offer mechanistic insight into why this topography changes through quantifying the fitness effect of individual mutations, and patterns of epistasis, across drug environments. Finally, we simulate evolution to observe how subtle differences in the topography of these otherwise cross-resistant landscapes create surprisingly different dynamics. We discuss the results in terms of their implications for the general study of empirical adaptive landscapes, in the context of more detailed models for the evolution of drug resistance, and with regards to how they refine our understanding of the cross-resistance concept.

## Methods

### System of study

**Fig 1:**
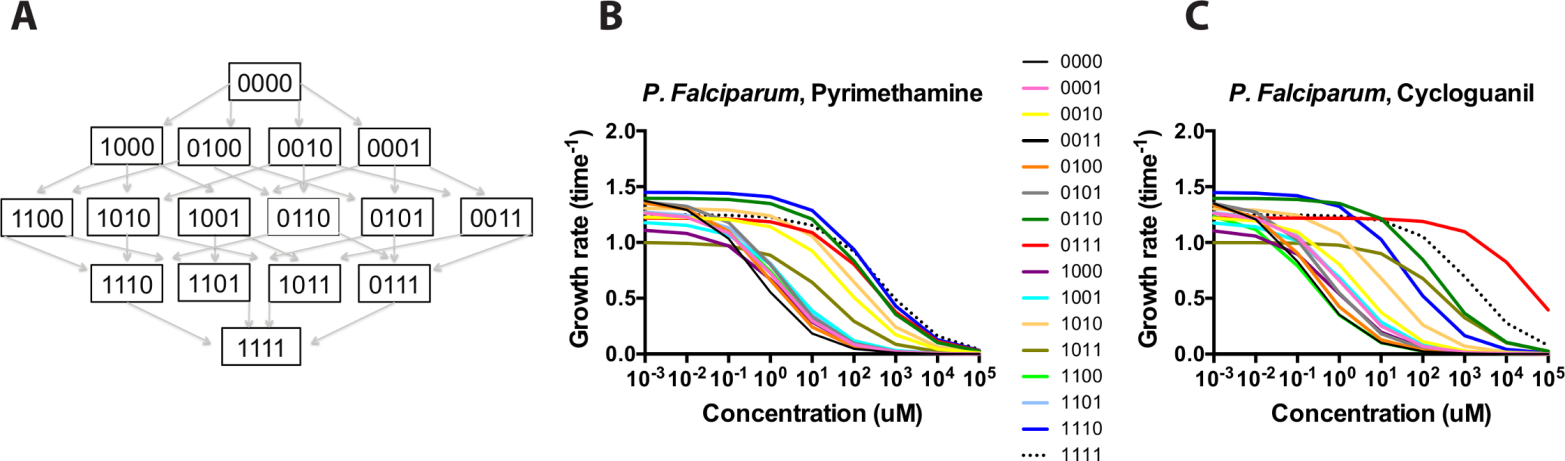
*P*. *falciparum* resistance to pyrimethamine (PYR) and cycloguanil (CYC): Basic structure of the adaptive landscape and growth rates across drug environment. (A) Basic structure of available pathways between the ancestor (0000) and quadruple mutant (1111). 0000 corresponds to the wild-type DHFR genotype, with 1111 the mutated protein at each of four sites (N51I, C59R, S108N, and I164L). (B, C) Growth curves for the *P*. *falciparum* alleles in pyrimethamine and cycloguanil, simulated from previously measured empirical measurements [11,12]. Growth rates are in terms of time^-1^. The *x*-axis is in terms Log_10_ of the drug concentration (uM). Note the subtle differences between the growth rate curves for the individual alleles across the two related drugs.

The study utilized data from a well-characterized system: transgenic *Sacharomyces cerevesiae* carrying a combinatorially complete set of resistance mutations for *P*. *falciparum* Dihyrofolate Reductase (DFHR)[11,12]. By “combinatorially complete,” we mean all combinations of mutations at the following sites corresponding to mutations identified in field isolates of *Plasmodium falciparum* in various settings [32–43]: N51I, C59R, S108N, I164L. We use bit string notation to represent the 16 alleles being studied, with 0000 corresponding to the wild type ancestor, and 1 to a mutation at each site (the 1111 allele the quadruple mutant). We also use asterisk notation to denote classes of alleles containing individual sites: 1*** (N51I), *1** (C59R), **1* (S108N), ***1 (I164L). Fig 1 shows the entire set of mutants for the landscape connected in all combinations between the ancestor (0000) and the quadruple mutant (1111). The empirical data—growth measurements without drug and IC_50_ values for all 16 alleles in pyrimethamine (PYR) and cycloguanil (CYC)—were measured in prior studies [11,12] and used to develop the more detailed model of evolution presented in this study.

### From empirical data to estimated growth rate, *g*

Following the literature, we assumed that growth rate is related to drug concentration via a logistic function with two empirically-derived parameters: the drugless growth rate and the IC_50_ (see S1 Table), both measured in previous studies using a transgenic yeast system [11,12]. Logistic curves were generated from an equation:

$$g\left(x\right)=\frac{g_{drugless}}{1+e^{\frac{{\text{IC}}_{50}\;\text{-}\;\text{x}}{c}}}$$ (1)

Where *g*_*drugless*_ is the growth rate with no drug present, the IC_50_ the concentration of drug that inhibits growth rate by 50%, and *c* a fitted constant that defines the shape of the logistic curve. The final growth rates were computed by standardizing the original drugless growth rates (in optical density) relative to the slowest-growing viable allele, 1011, which was given a *g*_*drugless*_ value of 1.0 (the 0011 allele has undetectable growth in this system, and is given a growth rate of 0). We determined growth rates across a window of pyrimethamine and cycloguanil concentrations between 0 and 100,000 uM, a range that includes blood levels of pyrimethamine measured within infected humans [44–48]. Drug concentrations are log transformed, and are represented in this study as Log [Drug] in micromolar (uM) concentration (S2 Table).

### On standard error in parameter measurements

Error estimates for growth rates at each drug concentration are unavailable because the estimated growth rates are modeled from Eq 1, using the empirically measured *g*_*drugless*_ and the IC_50_. We do, however, have standard errors for both of these parameters, which are quite small. These estimates have been published previously[11,12], and are reproduced in S1 Table. For the drugless growth rate, the standard errors range between 1% and 8%. For IC_50_ values, the standard errors are even lower, ranging from 0.2% to 3% in pyrimethamine and 0.4% and 4% in cycloguanil. We will further examine the role of experimental noise in the section dedicated to the fitness effects of individual mutations below.

### Pleiotropy across environments: Cross-resistance

Pleiotropy is broadly defined by a single genotype’s (an allele or mutation) effect on two or more phenotypes. Because we are examining landscapes in two different drugs, we sought to test pleiotropy more directly by determining whether the structures of the adaptive landscapes for the two drugs were consistent with “cross-resistance” as commonly understood. We assessed this in terms of (1) the correlation between the IC_50_ values for the alleles in landscapes of pyrimethamine and cycloguanil, and (2) the correlation between the growth rates of the alleles in pyrimethamine and cycloguanil across a range of drug concentrations.

### Effect of mutation as a function of the environment

To measure the effects of environment on distinct mutations, we utilized a novel method used to compute the change in fitness when each of a set of single mutations (the four sites examined in this study) was added to all possible genetic backgrounds [49]. Our version of this calculation is analogous to measures of gene by gene by environment interactions [50], or how much the effect of a mutation depends on genetic background and environment [20,51]. The number of mutational backgrounds per mutation can be calculated as follows: number of variants per site^(number of total sites—1)^ = 2^3^ = 8 possible genetic backgrounds.

The effect of a mutation can be computed by taking the difference between the fitness (*W*) of an allele *j* and the one-step neighbor carrying mutant *j*ε (N51I, C59R, S108N, I164L):

$$\Delta W_{\varepsilon }=W_{j_{\varepsilon }}-W_{j}$$ (2)

We computed the absolute effects of individual mutations on growth rate at each of the four sites across the measured drug concentrations. In addition, we calculated a scaled effect of each mutation by dividing the absolute effect on growth rate by the growth rate of the wild type ancestor (0000) at a given drug concentration. This relative contribution is important to highlight because we would like to identify those environments where the absolute effects of a mutation are large, but which have little effect on dictating evolutionary dynamics because all mutants have high fitness. Alternatively, we would also like to identify those environments where the absolute effect of a given mutation might be small but very consequential in evolutionary dynamics because they are large relative to the ancestor (e.g., 0000).

#### Quantifying epistasis

Above we describe how to measure the fitness impact of a given mutation on the fitness of an allele. Embedded in this concept is epistasis, recently defined as the “surprise at the phenotype when mutations are combined, given the constituent mutations’ individual effects [49].” While there are many different ways to quantify epistasis, we measure it by the standard deviation in fitness effect of a mutation in an environment. Measures of statistical dispersion (like standard deviation) are an appropriate proxy for epistasis because they capture how the fitness effects of mutations depend on genetic background, which underlies the “surprise” in the epistasis definition provided above.

### Simulations of evolution across drug environments

While previous studies of adaptive landscapes have identified probable pathways in evolution [3,11,13,14], none include information on dynamic properties of this evolution, how alleles rise and fall through frequency space. This is a notable omission, as only through studying dynamics can we observe how the rate of fixation and other dynamic properties depend on the environment. To test how the aforementioned changes in adaptive landscape structure affect the dynamics of evolution, we modeled a discrete (non-overlapping) generation, individual-based scheme using SimuPop, a forward-time simulation package [52]. Each run began with a population fixed for the 0000 ancestor, with a population size of 10^4^,where mutation and reproduction were probabilistic, rather than deterministic. The mutation rate was based on a normalized mutation matrix for *P*. *falciparum* as in Lozovsky et al., and adjusted for a per-site, per generation mutation rate. Finally, these mutation rates were scaled by a factor *m* (set to 10^3^), which allowed us to run much shorter simulations with a more manageable numbers of individuals, while not changing the qualitative results of simulations with much larger population sizes, as in Jiang et al (2013). We do this by dividing the effective population size by the scaling factor, *m*, and then multiplying all rates by that same factor:

$$N_{e}\cdot \mu =\frac{N_{e}}{m}\left(\mu \cdot m\right)$$ (3)

Where *N*_*e*_ is the effective population size, *μ* the mutation rate, and *m* the scaling factor.

Unlike Jiang et al. (2013), we modeled a starting population size that was identical in all simulation runs, at several drug concentrations: no drug, 1.0 uM, 100 uM and 10,000 uM. This static drug, long-term forward evolution scenario is analogous to a pathogen being treated consistently with a certain concentration of drug over a long duration. We ran 100 replicate simulations for each scenario, amounting to approximately 700 simulations: 1 no drug simulation, 3 pyrimethamine concentrations, 3 cycloguanil concentrations. Rather than simply reporting the “winning” allele or most frequently traversed trajectory, we were interested in the dynamics of evolution, and included illustrative examples of evolutionary simulations. In addition, we compared mean fixation times for alleles in the most preferred pathways across simulated environments.

## Results

### Growth rates

Fig 1 illustrates the general structure of the landscape and individual growth rates for its 16 alleles as a function of pyrimethamine and cycloguanil concentration. Note that the growth rate of the most resistant allele in pyrimethamine as judged by IC_50_ (1111) is the highest only at extreme drug concentrations, meaning that it is not uniformly favored despite its superior resistance (as measured by IC_50_; S1 Table). For cylcoguanil, the rank order of fitness values is different than for pyrimethamine, with the 0111 triple mutant having the highest growth rate across most drug concentrations. More broadly, we can observe how the rank order of growth rates varies across the range of drug concentrations and changes the topography of adaptive landscapes. We list the rank orders of alleles in **Table 1.**

**Table 1:** The rank order of fitnesses changes as a function of drug concentration. Here we observe how the rank order of the fitnesses of alleles across the landscape changes as a function of drug environment. This dynamic order implies that the probability of individual trajectories must also change as a function of drug. Alleles that are in **bold** correspond to those that have been consistently observed in field isolates of *P*. *falciparum* (See Supporting Information for more references). Also note differences between the rank order patterns of PYR and CYC. Columns with a 16 in parentheses indicate that the allele is actually tied for the lowest ranking of all alleles in the set for that environment.

	Pyrimethamine concentration (uM)									
Allele	0	10^−3^	10^−2^	10^−1^	1.0	10	10^2^	10^3^	10^4^	10^5^
**0000**	2	3	6	13	15	15	15	15	12(16)	8 (16)
0001	8	8	9	11	12	12	12	10	12 (16)	8 (16)
**0010**	11	11	11	5	6	6	6	6	6	7
0011	16	16	16	16	16	16	16	16	12 (16)	8 (16)
0100	4	5	4	9	14	14	14	10	12 (16)	8 (16)
0101	4	4	3	7	9	10	10	10	8	8 (16)
**0110**	2	2	2	2	2	2	3	4	4	2
**0111**	12	12	11	5	5	4	4	3	3	2
1000	14	14	14	14	13	13	12	10	12 (16)	8 (16)
1001	13	13	13	12	9	8	8	8	8	8 (16)
**1010**	6	6	4	3	3	5	5	5	5	5
1011	15	15	15	14	7	7	7	7	7	6
1100	8	8	9	10	11	11	11	10	8	8 (16)
1101	7	7	8	8	8	9	9	8	8	8 (16)
**1110**	1	1	1	1	1	1	1	2	2	2
**1111**	10	10	7	4	4	3	2	1	1	1
	**Cycloguanil concentration (uM)**									
**Allele**	**0**	**10** ^**−3**^	**10** ^**−2**^	**10** ^**−1**^	**1.0**	**10**	**10** ^**2**^	**10** ^**3**^	**10** ^**4**^	**10** ^**5**^
**0000**	2	3	9	14	13	14	15	11	8 (16)	8 (16)
0001	8	8	9	8	9	10	9	8	8 (16)	8 (16)
**0010**	11	11	11	6	7	7	7	7	7	8 (16)
0011	16	16	16	16	14	16	16	16	8 (16)	8 (16)
0100	4	5	6	12	12	13	13	11	8 (16)	8 (16)
0101	4	3	4	9	10	12	11	11	8 (16)	8 (16)
**0110**	2	2	2	2	1	1	3	3	3	3
**0111**	12	11	6	5	4	1	1	1	1	1
1000	14	14	14	12	11	11	11	11	8 (16)	8 (16)
1001	13	13	12	10	8	8	8	8	8 (16)	8 (16)
**1010**	6	6	3	4	5	6	6	6	6	8 (16)
1011	15	15	15	11	6	5	4	4	4	3
1100	8	10	13	15	13	14	13	11	8 (16)	8 (16)
1101	7	7	6	7	9	9	9	8	8 (16)	8 (16)
**1110**	1	1	1	1	2	4	5	5	5	5
**1111**	10	9	5	3	3	3	2	2	2	2

### Pleiotropy across environments: Cross-resistance

**Fig 2:**
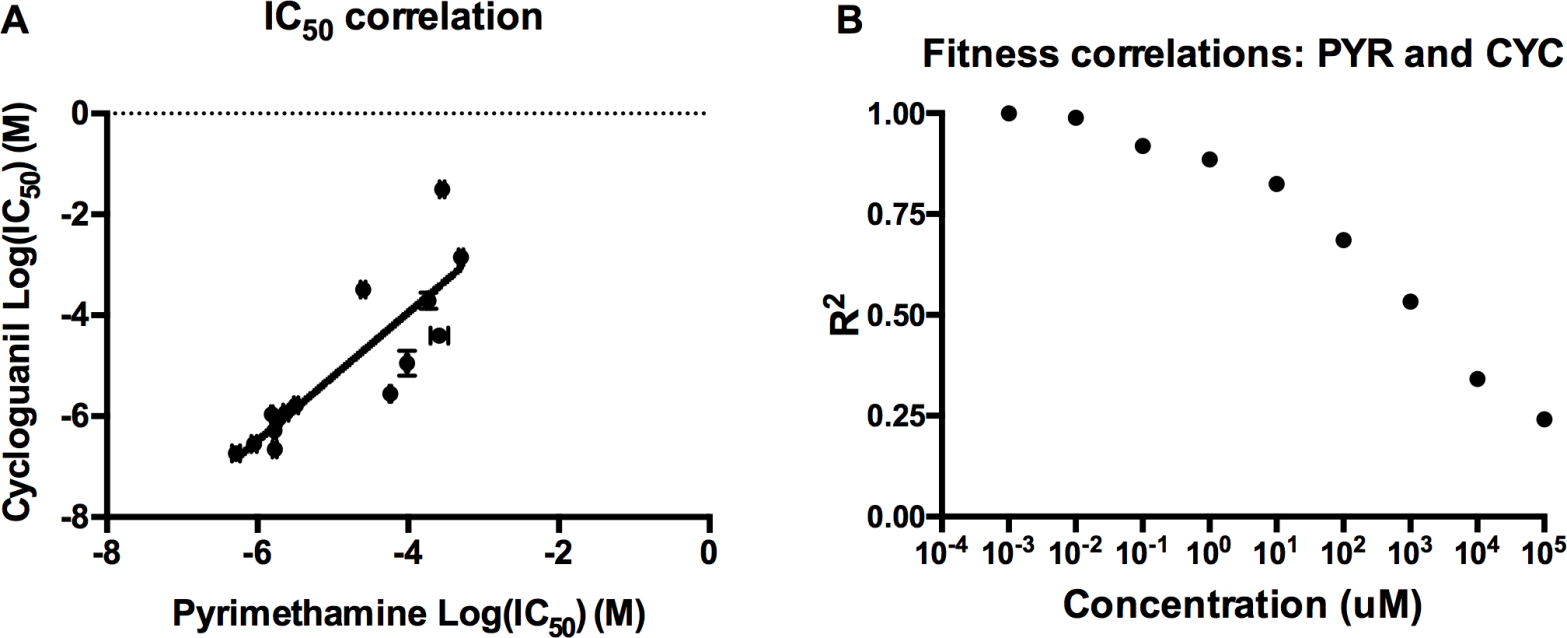
Resistance to pyrimethamine is correlated with resistance to cycloguanil. (A) Correlations between the IC_50_ values for pyrimethamine (*y*-axis) and cylcoguanil (*x*-axis). Each point corresponds to a single genotype in the landscape. The landscapes are significantly correlated for both traits, indicating cross-resistance (IC_50_: *R* ^2^ = 0.74, *P* = 3.9 X 10^−5^; Error bars on IC_50_ values represent standard errors of the published experimental replicates [11,12]. (B) A graph of the *R* ^2^ as a function of drug concentration, demonstrating that the landscapes remain correlated across drug concentrations, albeit less so as drug concentration increases. Pearson product-moment correlations were significant (*P* < 0.05) at all concentrations except the highest in our study (1.0 X 10^5^ uM), which was nearly significant (*P* = 0.067). Individual scatter plots that produce these *R* ^2^ values, and corresponding *P* values, can be found in S1 Fig.

Having demonstrated that the structure of adaptive landscape topography changes as a function of drug environment, we can address whether the 16 alleles that compose the adaptive landscapes for the two drugs demonstrate cross-resistance, that is, whether resistance phenotypes for one drug confer corresponding resistance to the other. To do this, we compared the landscapes with regards to their IC_50_ (a standard measure of resistance) and growth rates across all drug environments. Regression analysis of the IC_50_ values across the two drugs, observable in Fig 2A, yielded a significant correlation for IC_50_ (Pearson *R* ^2^ = 0.74, *P* = 3.9 X 10^−5^). In addition, the landscape showed significant or nearly significant correlations between the landscapes across drug concentrations (Fig 2B and S1 Fig), indicating that the landscapes share an essential structure. This is unsurprising, as pyrimethamine and cycloguanil are related compounds with a similar molecular structure and mechanism of action [53–55]. With that said, the decreasing correlation between landscapes with increasing drug concentration (Fig 2B and S1 Fig) suggests that cross-resistance is a quantity that may depend on the environment. Also note that this decrease in correlation could be an artifact of the overall decline in the growth rates of alleles as drug concentrations increase. Because more alleles have a growth rate close to 0 at high drug concentrations, the resolution in rates between alleles (necessary for a strong correlation) also declines. We mention this to highlight that the significant (or nearly significant) but declining correlations observed at higher concentrations might actually be stronger than the analysis and graphs communicate.

### Trajectory structure according to environment

**Fig 3:**
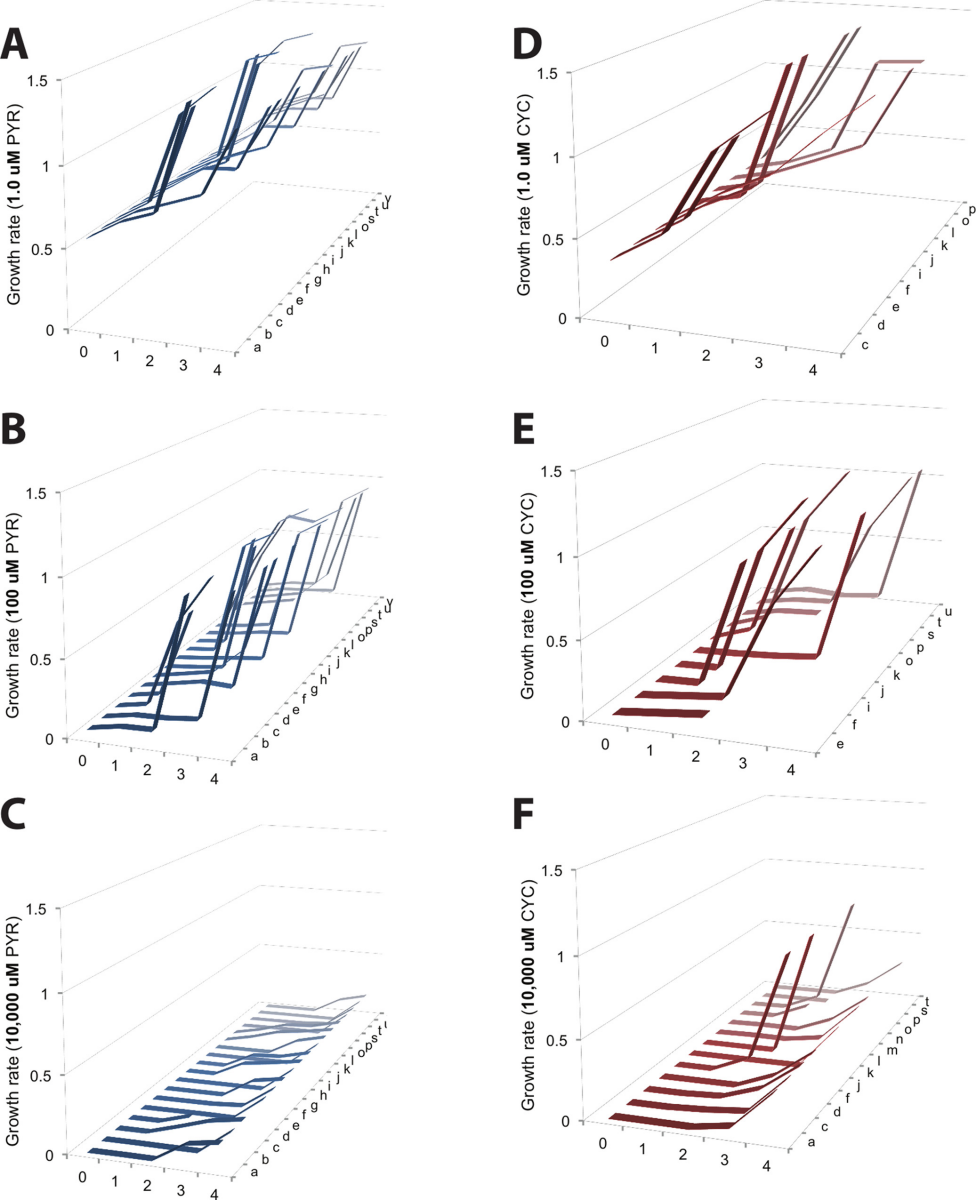
An illustration of how the structure of accessible adaptive trajectories differs between drug environments. Adaptive landscapes for *Plasmodium falciparum* across environments are organized into their accessible trajectories across several drug concentrations of pyrimethamine and cycloguanil. We define an accessible pathway as having an increasing fitness along the steps of a pathway. The five panels correspond to PYR (A-C, in blue) and CYC (D-F, in red). The *y*-axis is growth rate. The *x*-axis denotes Hamming distance (0 = wild type ancestor, 1 = single mutants, 2 = double mutant, 3 = triple mutant, 4 = quadruple mutant), and the *z*-axis corresponds to the 24 different possible pathways between the wild type ancestor (0000) and maximal resistant allele (1111) (see S3 Table for pathways a-x, corresponding to each individual pathway). Note how trajectories differ as a function of drug concentration (top to bottom) and drug type (left to right). Growth rates are in terms of time^-1^. Concentrations: no drug (A,D), 100 uM (B, E), and 10,000 uM (C,F). Note how both the number and topography of accessible pathways vary across drug environments.

Using fitness values for *P*. *falciparum* (computed using Eq 1), we reconstructed a 3-dimensional representation of all accessible trajectories between the ancestor (0000) and quadruple mutant (1111) across drug environments (Fig 3). We define a pathway as accessible if growth rate increases with Hamming distance, as in several related studies of adaptive landscapes [3,11,14]. Note that many such pathways will reach a fitness peak prior to the quadruple mutant, at a double or triple mutant state. In this figure, we observe how the environment has gross effects on evolution, affecting both the number of accessible pathways, and their topography. In particular, note subtle differences between the pyrimethamine and cycloguanil environments: There are fewer accessible pathways in cycloguanil than pyrimethamine in the three drug concentrations observed in Fig 3A vs. 3D; Fig 3B vs. 3E; Fig 3C vs. 3F.

With regards to epistasis: In the null expectation (i.e., trajectories without epistasis), the fitness would increase monotonically along each mutational pathway. That the fitness increases non-monotonically along some pathways is suggestive of magnitude epistasis, where fitness effects are not the same on all backgrounds. Sign epistasis underlies many non-accessible pathways (not observed in Fig 3) where there are fitness decreases with increased Hamming distance, constraining certain trajectories and making others more accessible [6,15]. We explore this in detail later in our study.

### Effect of mutation as a function of the environment: Mean fitness effect

To explain the ruggedness in adaptive landscape topography (Fig 3) and the observed patterns of cross-resistance (Fig 2), we calculated the average effect of individual mutations on reproductive fitness, across drug environments. To do so, we compared the effect of each mutation at the four candidate sites—N51I (1***), C59R (*1**), S108N (**1*), and I164L (**1*)—in both pyrimethamine and cycloguanil, across concentrations. We carried out this comparison for both the absolute growth rate effect and a rescaled measure that calculates the effect relative to the average growth rate in a given environment (see: **Methods**). The former informs us of how an environment dictates the total fitness effects of mutations. The latter (scaled effects) illuminates the picture relative to the growth rate of the wild type ancestor (0000). This is a critical distinction, because some mutations of low absolute effect might be very consequential for evolution in certain environments. At high drug concentrations (PYR and CYC), for example, all alleles have a low absolute growth rate. Nevertheless, alleles experience intense competition at these high drug concentrations because of meaningful differences in their *relative* growth rates.

**Fig 4:**
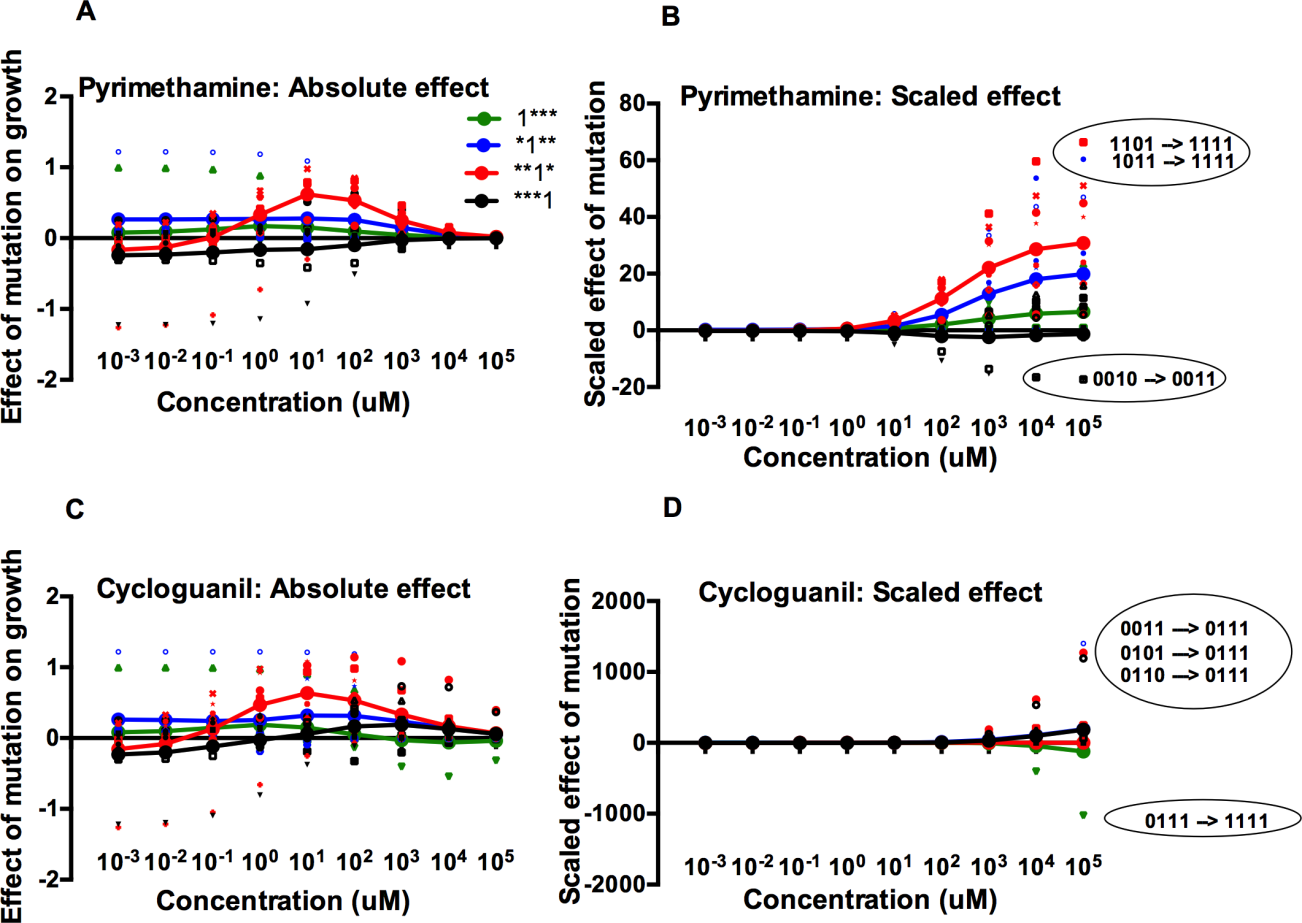
The effect of mutations changes as a function of drug environment and genetic background, with prominent epistasis. Each color represents the effect of a particular mutation. Small (unconnected) symbols represent individual mutational effects in a particular genetic background; large symbols (connected by lines) represent the average mutational effects over all genetic backgrounds. The scatter of the smaller symbols around the large ones indicates that individual effects are dependent on genetic background, the signature of epistasis. (A) and (B) represent the calculations for pyrimethamine (absolute and scaled, respectively), with (C) and (D) representing the calculations for cycloguanil. For 4B and 4D, units are in relative effects of mutation, a property determined by the absolute fitness effect divided by the growth rate of the ancestor (0000). (B) and (D) are also marked with circles and labels that identify especially large epistatic interactions (labeled in terms of the mutational events that produce the notable fitness effect) at particular drug concentrations. Due to the large differences in scaled effects for the two drugs, the *y*-axes for B and D have different ranges. Though epistasis can be observed here, the standard deviation in fitness effects was measured directly and plotted in S2 Fig. In 4D, the 0011→0111 mutation effect is slightly offset (vertically) so that it can be distinguished from the 0101→0111 mutation effect.

The shape of the curves in Fig 4 indicates an interaction between fitness effect and environment. The results of the formal analysis (ANOVA) of how the drug environment influences mutation effects is outlined in S4 Table and can be broadly summarized as follows: In pyrimethamine, the third site mutation (**1*) is most affected by environment (absolute: *F* = 4.57, df = 9,70, *P* = 0.00009; scaled: *F* = 8.10, df = 9,70, *P* = 4.0 × 10^−8^). This is especially true at high drug concentrations, as the third site mutation is present in the most resistant single mutant (0010), double mutant (0110), triple mutants (1110 and 0110) and the maximally resistant quadruple mutant (1111) (Fig 1B and Table 1).

In cycloguanil, the situation is different, and the mutation effect findings are dominated by the 0111 allele that is substantially more resistant (as measured by IC_50_, S1 Table) than any other in its adaptive landscape. For those reasons, all mutations that can generate 0111 have positive absolute effects for much of the breadth of drug concentrations, and especially the higher drug concentrations. The third site, **1*, has a statistically significant interaction with drug concentration (absolute effect: *P* = 0.00081) with the fourth site, ***1 having a nearly significant interaction across environments (absolute effect: *P* = 0.05). These findings relate to the role each site plays in creating not only the 0111 mutant, but also the most resistant double mutant (0110), and other mildly resistant triple mutants (1011).

#### Quantifying epistasis

**Fig 5:**
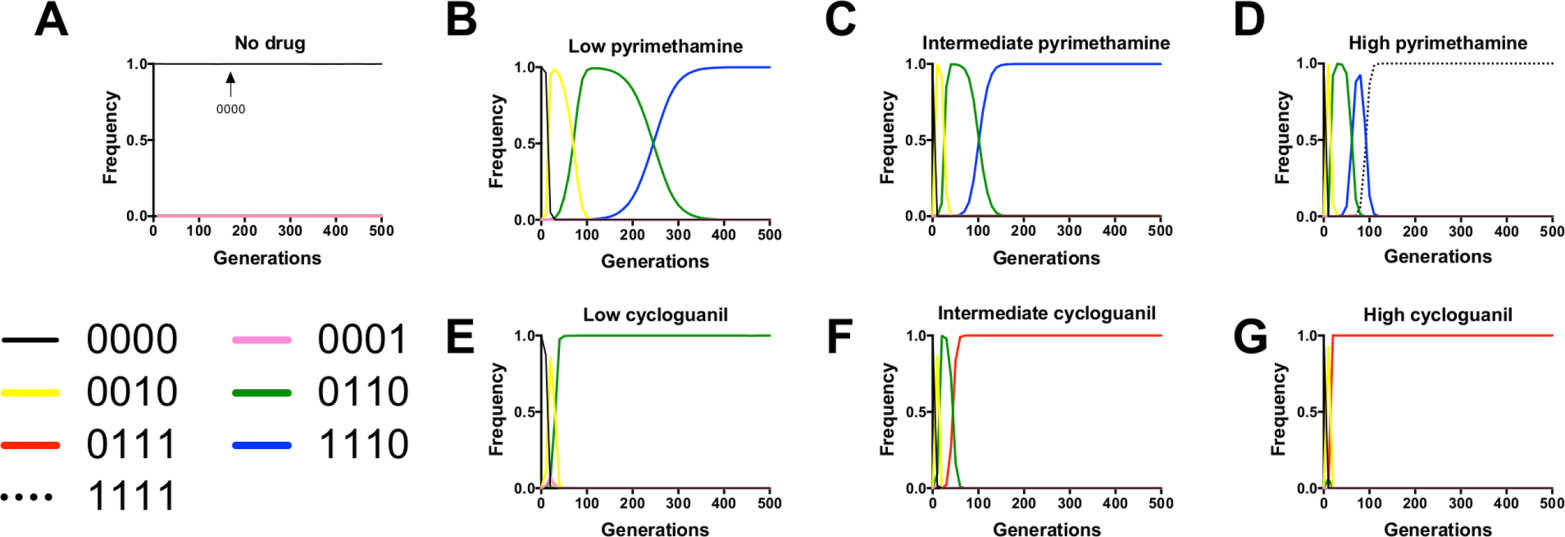
Simulations of evolution in pyrimethamine (PYR) and cycloguanil (CYC) reveal differences in evolutionary dynamics across environments. These are illustrative examples of the most preferred pathways for evolution at each of the simulated PYR and CYC environments. Panels correspond to several simulation scenarios: (A) no drug, (B) low PYR (1.0 uM), (C) intermediate PYR (100 uM), (D) high PYR (10,000 uM), (E) low CYC (1.0 uM), (F) intermediate CYC (100 uM), and (G) high CYC (10,000 uM). Here we observe 5 different fixing alleles across the 7 different drug environments. This is indicative of how the preferred adaptive trajectory is strongly dependent on drug environment. Not only do pathways differ, but the mean fixation times for the fixing allele also differs significantly across environment: *F* = 763.36, df = 5,564, *P* < 0.0000001; S6 Table).

Having analyzed the differences in the mean fitness effects of mutations, we next turn our attention to the dispersion around the mean values (the small, unconnected markers in Fig 5), which is indicative of prominent epistasis (sign and magnitude) between mutations and genetic backgrounds across drug environments. For example, a landscape without epistasis would show no scatter of points (other than that due to measurement error), as all substitutions would have the same fitness effect on all genetic backgrounds. S2 Fig demonstrates that the standard deviation of the fitness effects, a measure of dispersion and a proxy for epistasis, differs between the two drug environments, partially explaining the differences in landscape topography of as observed in Fig 3.

#### Ratio of experimental noise to mutation effects

In order to directly examine the relationship between the experimental noise and the mutation effects, we calculated the ratio between the standard error for the growth measurements and the standard error of mutation effects. This is modeled in the drugless environment because it contains the maximum growth rates for all alleles with empirical standard errors. From this analysis we observe that the majority of ratios are below 1.0, indicating that experimental noise is smaller than the variance in fitness effects between neighboring alleles on the simulated landscapes. See S5 Table for further discussion.

### Simulations of drug treatment

To observe how adaptive landscape by environment interactions affect evolutionary dynamics, we used growth rates derived from empirical data to simulate the evolution of populations at various drug concentrations (see: **Materials and Methods** for details). In this model, a population of 10,000 individuals fixed for the ancestor (0000) were exposed to single, stable concentrations of pyrimethamine or cycloguanil in several drug environments: no drug (Fig 5A), low (1.0 uM) (Fig 5B and 5E), intermediate (100 uM) (Fig 5C and 5F) and high (10,000 uM) (Fig 5D and 5G). The graphs represent illustrative examples of the most preferred pathways and typical evolutionary dynamics in each environment. We will discuss the main results of simulations in each drug environment, and provide a summary of all simulations in S6 Table (which also includes the results of statistical tests comparing the mean fixation times along preferred pathways).

#### No drug (Fig 5A)

When evolution was simulated in the no drug environment, it remained fixed for the 0000 ancestral genotypes through 1000 generations. Though the 0000 ancestor is not a fitness peak in the drugless environment, locating the higher fitness 1110 allele requires traversing an adaptive landscape that includes several lower fitness valleys (3A, 3D). Explanations for this are also apparent from Fig 4B and 4D, showing that the scaled fitness effects of all mutations are almost negligible at low drug concentrations, suggesting few mutational pathways from 0000 that would provide an accessible avenue towards 1110. Because of this, the population remains trapped in the ancestral (0000) state.

#### Pyrimethamine, low drug (1.0 uM) (Fig 5B)

At the low PYR environment, the 1110 triple mutant fixes, at approximately 370 generations on average, through the 0010 and 0110 intermediates. We observe each of the intermediates making very clear and prominent appearances in frequency space, reaching near fixation before giving way to their downstream mutant neighbors. The results illustrated in Fig 4B help to explain these results, where the high average fitness effect of the third site mutation (**1*) (which includes the creation of 0010 from 0000 and 0110 from 0100 at 1.0 uM PYR) creates “stepping stones” for the 1110 high fitness variant.

#### Pyrimethamine, intermediate drug (100 uM) (Fig 5C)

Similar to the low PYR environment, the 1110 triple mutant fixes through the 0010 and 0110 intermediates. The difference is that selection is more extreme at the intermediate PYR concentration, and so the fixation time is faster (average = 127.1 vs. 368.4; S6 Table).

#### Pyrimethamine, high drug (10,000 uM) (Fig 5D)

At high drug, the quadruple mutant 1111 fixes through the 0010, 0110, and 1110 intermediates, which have a brief but conspicuous appearance in frequency space. Note that in Fig 4B, the strongest epistatic interactions (1101 → 1111 and 1011 → 1111) both create the quadruple mutant, the most resistant as measured by IC_50_ (S1 Table).

#### Cycloguanil, low drug (1.0 uM) (Fig 5E)

Here we find fixation of the double mutant, 0110, the allele with the highest fitness of the set at 1.0 uM CYC, through a 0010 intermediate. We also consistently observe the ephemeral arrival of the 0001 single mutant that is unable to drive any downstream pathways and quickly disappears from frequency space.

#### Cycloguanil, intermediate drug (100 uM) (Fig 5F)

The triple mutant 0111 fixes before 200 generations through the 0010 and 0110 intermediates, both of which have brief but clear appearances in frequency space. From Fig 4D, note that several mutation events into 0111 have dramatically positive fitness effects, which is an indication of just how much better 0111 performs than any other allele in the landscape at intermediate and high CYC concentrations. Also note the decline in the fitness effect of the first site mutation (1***) (green line and circles) as drug concentration increases. This occurs because the 0111 high-resistance allele has no first site (N50I) mutation. Consequently, alleles carrying this first site mutation (absent in the high fitness 0111 allele) are relatively maladapted to the intermediate and high CYC environments.

#### Cycloguanil, high drug (10,000 uM) (Fig 5G)

The triple mutant 0111 fixes rapidly through the 0010 and 0110 intermediates, with both the 0010 and 0110 intermediate present so transiently as to be barely visible. This is consistent with a population genetic scenario where the 0111 triple mutant is so superior in growth to its 0010 and 0110 “stepping stones” that it displaces them in frequency space before they have risen to any appreciable frequency. This resembles the stochastic tunneling phenomenon in population genetics where intermediate genotypes are seemingly “skipped” during evolution under certain conditions [56,57].

## Discussion

Several recent examinations of adaptive landscapes have been conducted with regard to evolutionary genetics concepts like higher-order epistasis [49,58,59,60] or include mathematical descriptions of landscape topography [61,62]. While our study uses evolutionary and population genetics principles to explore landscape structure, it uses simulations to demonstrate how the environment influences the outcome of evolution and relates these dynamics to the fitness effects of individual mutations. This represents an extension of prior studies of adaptive landscapes, where resistance metrics like the IC_50_ or MIC were used to determine the likely pathway of evolution. Insofar as adaptive evolution in nature occurs in the presence of dynamic environments, this study provides a conceptual model for how dynamic evolution occurs across biological systems.

### Adaptive landscape topography varies drastically according to environment, which greatly affects the dynamics of evolution

Upon constructing a visual representation for discrete evolutionary pathways in *P*. *falciparum*, we observed that the overall topography of the landscape is strongly dependent on environment (Fig 3). The results of simulations highlight that dynamic properties of evolution, such as the identity of the most preferred pathway and fixation time of the highest fitness allele, are both dependent on the environment (Fig 5 and S6 Table).

Given the degree to which environmental circumstances affect evolution in our simulations, coupled with knowledge that *in vivo* drug concentrations vary within patients over time [44–48], we suggest that future characterizations of adaptive landscapes should be considered with respect to the full breadth of environments in which organisms exist. This suggestion is consistent with the more modern "fitness seascape" analogy [63], befitting a more dynamic model of evolution.

#### Observed pathways and alleles recapitulate findings from nature

In addition, it is important to note that our findings recapitulate findings in field settings, where the triple mutant containing N51I, C59R, and S108N (1110) has risen to high frequency in many settings where pyrimethamine-based therapies have been used (See **Supplementary Information** for references). Several intermediates observed in our simulations, including 0100, 0010, 0110, have been observed in field settings. While previous studies of adaptive landscapes for drug resistance have also returned preferred pathways composed of alleles found in nature, our work differs by proposing that double and triple mutants are more than simply “intermediates,” but rather, optimal alleles (fitness peaks) in certain drug environments (Figs 1 and 3 and 5, Table 1). We also demonstrate that selection for the quadruple mutant (1111) should only occur at very high drug concentrations (in pyrimethamine, and not at all in cycloguanil).

#### Epistatic interactions between certain mutations and genetic backgrounds drive the dynamics of evolution across environments

We utilized an existing method for the measure of mutation effects [49] but applied it to a situation where fitness was a function of drug environment. The quantification of how mutation effects change with environment allows us to make better sense of the evolutionary outcomes observed in our simulations. Generally, the third site (**1*, S108N) mutation has positive effects on fitness (in both pyrimethamine and cycloguanil), as it is present in several high fitness variants across a range of environments (0010, 0110, 0111, 0111, and 1111).

The non-significant results for the other mutations (1***, *1**, and ***1) (S4 Table) might be the more provocative finding, however, as they can be partially explained by the dispersion of individual mutation effect data points (the smaller individual markers in Fig 4) around the mean values (the larger markers connected with a line in Fig 4). This dispersion results from epistasis, where the fitness effects of mutations differ depending on the genetic background on which they occur (again: were there no epistasis, all individual mutation effects would be the same in magnitude, with none of the dispersion as observed in Fig 4). That we still see one mutation (**1*) with a consistently positive effect is, however, striking. We might describe this third site mutation as a “pivot mutation,” that, because of its reliably positive fitness effect across elevated drug concentrations, serves as a pivot point that directs evolution down certain pathways.

#### IC_50_ and MIC do not reliably predict how drug resistant alleles will perform at all relevant drug concentrations

In the simulations, we observed that in many scenarios the most resistant allele (as measured by IC_50_) was not favored, even under moderately high drug concentrations. This is unsurprising because the IC_50_ metric only speaks to how well a parasite will resist the effects of drug; if the parasite replicates poorly it can carry a high resistance phenotype and still be outcompeted in some drug concentrations. This same logic applies to the minimal inhibitory concentration (MIC), a metric that only accounts for complete growth inhibition and ignores growth characteristics across the range of drug concentrations that might include the average treatment environment. Said differently, use of the IC_50_ (or MIC) as a standard of drug resistance gives undue attention to the allele that is the most difficult to suppress the growth of. This is not the same as the allele most likely to cause a virulent infection, to be transmitted, or to initiate an epidemic at any particular drug concentration.

We can easily demonstrate how use of the IC_50_ as the sole resistance proxy can mislead our predictions by comparing the results of simulations of *P*. *falciparum* DHFR resistance evolution where IC_50_ was used as a fitness proxy [11,12,14] to the results from our study. In pyrimethamine, prior studies of probable adaptive trajectories showed the 0000 → 0010 → 0110 → 0111 → 1111 pathway to be the most observed [11], whereas this pathway arose in a negligibly small number of simulations in our current study at lower drug concentrations (S6 Table). The explanation is rather simple: the 0111 triple mutant intermediate, highly resistant by measures of IC_50_ (S1 Table), has a mediocre drugless growth rate, and is outgrown by the 1110 triple mutant at all of the pyrimethamine concentrations that we explored, even though 0111 has a higher resistance (IC_50_). In cycloguanil, the data from this study are a bit less divergent from those in the IC_50_ based study [12] as the most realized pathway is the same that we observe at intermediate and high drug cycloguanil concentrations in our study (Fig 5F and 5G). However, this is not true for the lower cycloguanil concentrations (Fig 5E) where the double mutant 0110 outcompetes the far more resistant 0111 triple mutant. From this, we can suggest that future studies of adaptive trajectories in drug resistance models should use caution in equating any singular measure of resistance with fitness across all plausible drug environments, as they are likely to misidentify likely pathways and trajectories at many drug concentrations lower than the most extreme.

Going forward, these findings propound an important general question about the utility of resistance metrics: if not the IC_50_ or MIC, what single summary statistic would be appropriate for predicting probable evolutionary trajectories? Our answer is that summary statistics are not always necessary and instead, one should use the actual fitnesses of alleles in the environments of interest. Were we to operationalize this understanding towards a simple method for predicting the likely direction of evolution (in situations where observing it directly is challenging), we might suggest that: (a) the researcher know the range of environments of interest, (b) the researcher know the fitness values of all alleles composing the landscape in these environments of interest and, if possible, (c) the researcher use a population genetic model (mathematical or computational) to identify probable pathways. In a situation where the entire growth curve is unavailable, we suggest that a researcher at least measure the drugless growth rate in addition to the IC_50_, and attempt to simulate growth curves based on empirical and estimated parameters as in Eq 1.

#### Standard definitions of “cross-resistance” are insufficient for understanding resistance patterns for drugs with multi-allele adaptive landscapes

Despite being similar in chemical structure, target of action [53–55], and in cross-resistance as commonly understood (Fig 2), pyrimethamine and cycloguanil produce different patterns of epistasis (Fig 4 and S2 Fig), and consequently, different *in silico* evolutionary dynamics (Fig 5 and S6 Table). That two similar drugs can produce such different results forces us to rethink the definition of cross-resistance. When we say that a pathogen variant exhibits cross-resistance to different drugs, what do we mean? That the mechanism of action is similar? That the adaptive landscapes corresponding to the two drug environments are similar? That pleiotropy and tradeoffs manifest similarly? If so, how similar? If two adaptive landscapes are deemed equivalent by some metric, what if we find that even slight differences in adaptive landscape topography lead to different evolutionary outcomes, as in this study? Our results highlight that these questions might have been taken for granted, and are important for understanding any microbe-drug system.

In rethinking this, we emphasize more quantitative measures of cross-resistance: instead of it being a descriptive term, cross-resistance between landscapes in the environments of interest should be defined by the actual correlation coefficient (*R* ^2^), as in some prior studies [64,65]. In our system, the magnitude of cross-resistance would be defined by the values as depicted in Fig 2B and S1 Fig. In doing so, we are keeping the essence of the cross-resistance concept intact, while stressing its quantitative dimensions, making it more appropriate for multi-allele adaptive landscapes.

#### Study limitations

This study is subject to the limitations of both model-system biology and evolutionary simulations. Regarding the former, the fitness values are derived from model system laboratory experiments and do not necessarily overlap with those in clinical infections. In the latter sense, the computational model's simplicity can be criticized on the grounds that it doesn’t provide a more literal analogue for clinical infection and treatment. Thankfully, the results can be improved upon with more clinically relevant contexts, including PK/PD [66,67] and spatial distribution models [68] that realistically track the concentration of drug in simulations. In addition, more rigorous clinical models should include parameterization for host biology (immunity and other properties) and behavior (adherence and side effects of drugs), all of which are natural extensions to our stricter evolutionary genetics examination.

#### Towards more refined models for the evolution of antimicrobial resistance (and beyond)

While our study was confined to a single locus (DHFR), and used simulations of empirical data derived from a model system, many of the approaches—the mapping of probable pathways, the quantification of pleiotropy (cross-resistance), the computing of changes in mutation effect and epistasis—are applicable to many biological systems. Any system where phenotypes can be determined for a combinatorially complete set of mutants across a breadth of environments can be understood in terms of adaptive landscape by environment interactions. This includes other infectious diseases, cancers of several kinds and large-scale ecological problems such as those in conservation biology, where a major challenge is keeping track of how genetic variation in populations will respond to changes in the environment.

## Supporting Information

S1 Fig Scatterplots depicting the correlations between growth rates of the 16 alleles for the two drugs (PYR and CYC) at several drug concentrations. *P* values correspond to the significance of correlations determined by the Pearson Product-Moment test.
(DOCX)

S2 Fig Quantifying epistasis. From Fig 4 we can plot the total dispersion in fitness effect as a function of drug environment, which provides a proxy for epistasis, as this dispersion is indicative of how the effect of a mutation depends on genetic background (G X G). This is graph of the standard deviation for each of the four mutations as described in the main text. We include both the standard deviation for the absolute and scaled effects.
(DOCX)

S1 Table Values and standard errors for the empirical derived parameters used to model growth rates: Drugless growth rates, IC_50_ values. Values for the allele 0011 were omitted, as it has a drugless growth rate below the detection limit, and consequently, has no means of determining an IC_50_. *Allele 0111 was resistant beyond the detection limit of the system in which its resistance was measured. That the 0111 resistance was so high as to be beyond the detection limit of the assay in which it was measured doesn’t affect the qualitative results, as it is the most preferred allele for the majority of CYC environments examined in this study. See main text and references for details.
(DOCX)

S2 Table Simulated growth rates. These are the growth rates generated from empirical parameters as discussed in the Methods section.
(DOCX)

S3 Table Discrete pathways with letter corresponding to the paths in Fig 3. (DOCX)

S4 Table ANOVA: Interaction between mutation effect and drug concentration for both pyrimethamine and cycloguanil. All values for df _num., denom._ = 9, 70.
(DOCX)

S5 Table Ratio of experimental noise to variance in fitness effects. This analysis directly investigates the ratio between experimental noise and the dispersion of fitness effects for all measurable alleles in the drugless environment (where fitness values are the highest for all alleles). The table indicates that for 14/15 alleles with measured fitness values, the standard error in the fitness measurements is smaller than the standard error of the fitness effects of mutations (ratio < 1.0), justifying the study of the landscape in terms of discrete alleles.
(DOCX)

S6 Table Summary of simulations of evolution across several drug concentrations. Average times to fixation are displayed for major pathways, which demonstrates how the timescale of evolution changes as a function of environment. ANOVA results compare the fixation times for preferred pathways a-g.
(DOCX)

S1 References This is a list of references that corresponds to findings from field studies of *P*. *falciparum* DHFR mutations with varying resistance to pyrimethamine. (DOCX)
