# Supplementary material for: Adaptive Landscape by Environment Interactions Dictate Evolutionary Dynamics in Models of Drug Resistance
Source: PLoS Comput Biol. 2016 Jan 25;12(1):e1004710. doi: 10.1371/journal.pcbi.1004710 (PMC4726534; doi:10.1371/journal.pcbi.1004710)
Supplement: S1 Fig — P values correspond to the significance of correlations determined by the Pearson Product-Moment test. (DOCX) [file pcbi.1004710.s001.docx]

In Figure 2, we showed how the IC50 and R^2^ values for the fitness at each drug concentration demonstrated at least reasonably strong correlation across drug concentrations. Here we show the individual correlations that were used to create Figure 2B, which are the fitness values for the landscapes for pyrimethamine and cycloguanil across drug concentrations. *P* values for correlations are reported on the graph, below the title.

**S1 Fig**. **Scatterplots depicting the correlations between growth rates of the 16 alleles for the two drugs (PYR and CYC) at several drug concentration.** *P* values correspond to the significance of correlations determined by the Pearson Product-Moment test.
