# Supplementary material for: Adaptive Landscape by Environment Interactions Dictate Evolutionary Dynamics in Models of Drug Resistance
Source: PLoS Comput Biol. 2016 Jan 25;12(1):e1004710. doi: 10.1371/journal.pcbi.1004710 (PMC4726534; doi:10.1371/journal.pcbi.1004710)
Supplement: S2 Fig — From Fig 4 we can plot the total dispersion in fitness effect as a function of drug environment, which provides a proxy for epistasis, as this dispersion is indicative of how the effect of a mutation depends on genetic background (G X G). This is graph of the standard deviation for each of the four mutations as described in the main text. We include both the standard deviation for the absolute and scaled effects. (DOCX) [file pcbi.1004710.s002.docx]

**S2 Figure. Standard deviation of mutation effect (epistasis)**

From Figure 4 we can plot the total dispersion in fitness effect as a function of drug environment, which provides a proxy for epistasis, as this dispersion is indicative of how the effect of a mutation depends on genetic background (G X G). Here is a plot of the standard deviation of the fitness effect for each of the four mutations as described in the main text. We include the standard deviations for both the absolute and scaled fitness effects.
