# Supplementary material for: Adaptive Landscape by Environment Interactions Dictate Evolutionary Dynamics in Models of Drug Resistance
Source: PLoS Comput Biol. 2016 Jan 25;12(1):e1004710. doi: 10.1371/journal.pcbi.1004710 (PMC4726534; doi:10.1371/journal.pcbi.1004710)
Supplement: S1 Table — Values for the allele 0011 were omitted, as it has a drugless growth rate below the detection limit, and consequently, has no means of determining an IC50. *Allele 0111 was resistant beyond the detection limit of the system in which its resistance was measured. That the 0111 resistance was so high as to be beyond the detection limit of the assay in which it was measured doesn’t affect the qualitative results, as it is the most preferred allele for the majority of CYC environments examined in this study. See main text and references for details. (DOCX) [file pcbi.1004710.s003.docx]

| Empirical Parameters | | | | | | |
| --- | --- | --- | --- | --- | --- | --- |
| Allele | Drugless Growth | S.E. | Log (IC_50_) Pyrimethamine | S.E. | Log (IC_50_) Cycloguanil | S.E. |
| 0000 | 1.398 | 0.0535 | -6.286 | 0.053 | -6.741 | 0.036 |
| 0001 | 1.275 | 0.0131 | -5.812 | 0.013 | -5.955 | 0.038 |
| 0010 | 1.227 | 0.0195 | -4.239 | 0.014 | -5.562 | 0.023 |
| 0100 | 1.370 | 0.0287 | -6.046 | 0.035 | -6.548 | 0.049 |
| 0101 | 1.375 | 0.0164 | -5.774 | 0.019 | -6.282 | 0.071 |
| 0110 | 1.397 | 0.0268 | -3.732 | 0.025 | -3.706 | 0.163 |
| 0111 | 1.219 | 0.0737 | -3.55 | 0.033 | -1.5^*^ | - |
| 1000 | 1.119 | 0.0349 | -5.724 | 0.029 | -6.049 | 0.022 |
| 1001 | 1.184 | 0.0595 | -5.491 | 0.029 | -5.778 | 0.077 |
| 1010 | 1.306 | 0.0336 | -4.015 | 0.017 | -4.947 | 0.248 |
| 1011 | 1.000 | 0.0814 | -4.6 | 0.033 | -3.494 | 0.08 |
| 1100 | 1.273 | 0.0509 | -5.773 | 0.028 | -6.663 | 0.041 |
| 1101 | 1.282 | 0.0444 | -5.624 | 0.034 | -5.925 | 0.028 |
| 1110 | 1.450 | 0.0159 | -3.587 | 0.116 | -4.395 | 0.049 |
| 1111 | 1.250 | 0.0457 | -3.3 | 0.033 | -2.85 | 0.118 |

**S1 Table.** **Values and standard errors for the empirical derived parameters used to model growth rates: Drugless growth rates, IC_50_ values.** Values for the allele 0011 were omitted, as it has a drugless growth rate below the detection limit, and consequently, has no means of determining an IC_50_. ^*^Allele 0111 was resistant beyond the detection limit of the system in which its resistance was measured. See main text and references for details.
