# Supplementary material for: Adaptive Landscape by Environment Interactions Dictate Evolutionary Dynamics in Models of Drug Resistance
Source: PLoS Comput Biol. 2016 Jan 25;12(1):e1004710. doi: 10.1371/journal.pcbi.1004710 (PMC4726534; doi:10.1371/journal.pcbi.1004710)
Supplement: S2 Table — These are the growth rates generated from empirical parameters as discussed in the Methods section. (DOCX) [file pcbi.1004710.s004.docx]

Pyrimethamine concentration (uM)

| **Alleles** | **0** | **10^-3^** | **10^-2^** | **10^-1^** | **1.0** | **10** | **10^2^** | **10^3^** | **10^4^** | **10^5^** |
| --- | --- | --- | --- | --- | --- | --- | --- | --- | --- | --- |
| **0000** | 1.40 | 1.37 | 1.29 | 1.03 | 0.55 | 0.18 | 0.05 | 0.01 | 0.00 | 0.00 |
| 0001 | 1.27 | 1.26 | 1.23 | 1.08 | 0.72 | 0.30 | 0.08 | 0.02 | 0.00 | 0.00 |
| **0010** | 1.23 | 1.23 | 1.22 | 1.21 | 1.14 | 0.92 | 0.51 | 0.17 | 0.04 | 0.01 |
| 0011 | 0.00 | 0.00 | 0.00 | 0.00 | 0.00 | 0.00 | 0.00 | 0.00 | 0.00 | 0.00 |
| 0100 | 1.37 | 1.35 | 1.30 | 1.10 | 0.66 | 0.24 | 0.07 | 0.02 | 0.00 | 0.00 |
| 0101 | 1.37 | 1.36 | 1.32 | 1.18 | 0.80 | 0.33 | 0.10 | 0.02 | 0.01 | 0.00 |
| **0110** | 1.40 | 1.40 | 1.39 | 1.39 | 1.35 | 1.21 | 0.83 | 0.36 | 0.10 | 0.03 |
| **0111** | 1.22 | 1.22 | 1.22 | 1.21 | 1.19 | 1.09 | 0.80 | 0.38 | 0.11 | 0.03 |
| 1000 | 1.12 | 1.11 | 1.08 | 0.97 | 0.67 | 0.29 | 0.08 | 0.02 | 0.00 | 0.00 |
| 1001 | 1.18 | 1.18 | 1.15 | 1.07 | 0.80 | 0.39 | 0.12 | 0.03 | 0.01 | 0.00 |
| **1010** | 1.31 | 1.31 | 1.30 | 1.29 | 1.24 | 1.06 | 0.65 | 0.24 | 0.06 | 0.02 |
| 1011 | 1.00 | 1.00 | 0.99 | 0.97 | 0.89 | 0.64 | 0.29 | 0.09 | 0.02 | 0.01 |
| 1100 | 1.27 | 1.26 | 1.23 | 1.09 | 0.74 | 0.31 | 0.09 | 0.02 | 0.01 | 0.00 |
| 1101 | 1.28 | 1.27 | 1.24 | 1.13 | 0.81 | 0.37 | 0.11 | 0.03 | 0.01 | 0.00 |
| **1110** | 1.45 | 1.45 | 1.45 | 1.44 | 1.41 | 1.29 | 0.94 | 0.43 | 0.13 | 0.03 |
| **1111** | 1.25 | 1.25 | 1.25 | 1.24 | 1.23 | 1.15 | 0.92 | 0.49 | 0.16 | 0.04 |

Cycloguanil concentration (uM)

| **Alleles** | **0** | **10^-3^** | **10^-2^** | **10^-1^** | **1.0** | **10** | **10^2^** | **10^3^** | **10^4^** | **10^5^** |
| --- | --- | --- | --- | --- | --- | --- | --- | --- | --- | --- |
| **0000** | 1.40 | 1.35 | 1.21 | 0.83 | 0.35 | 0.10 | 0.02 | 0.01 | 0.00 | 0.00 |
| 0001 | 1.27 | 1.26 | 1.21 | 1.05 | 0.66 | 0.25 | 0.07 | 0.02 | 0.00 | 0.00 |
| **0010** | 1.23 | 1.22 | 1.19 | 1.09 | 0.80 | 0.37 | 0.11 | 0.03 | 0.01 | 0.00 |
| 0011 | 0.00 | 0.00 | 0.00 | 0.00 | 0.00 | 0.00 | 0.00 | 0.00 | 0.00 | 0.00 |
| 0100 | 1.37 | 1.33 | 1.22 | 0.90 | 0.42 | 0.13 | 0.03 | 0.01 | 0.00 | 0.00 |
| 0101 | 1.37 | 1.35 | 1.27 | 1.02 | 0.55 | 0.18 | 0.05 | 0.01 | 0.00 | 0.00 |
| **0110** | 1.40 | 1.40 | 1.39 | 1.39 | 1.35 | 1.21 | 0.85 | 0.37 | 0.11 | 0.03 |
| **0111** | 1.22 | 1.22 | 1.22 | 1.22 | 1.22 | 1.21 | 1.19 | 1.10 | 0.82 | 0.40 |
| 1000 | 1.12 | 1.10 | 1.06 | 0.90 | 0.54 | 0.20 | 0.05 | 0.01 | 0.00 | 0.00 |
| 1001 | 1.18 | 1.17 | 1.14 | 1.01 | 0.69 | 0.29 | 0.08 | 0.02 | 0.00 | 0.00 |
| **1010** | 1.31 | 1.30 | 1.29 | 1.24 | 1.08 | 0.68 | 0.26 | 0.07 | 0.02 | 0.00 |
| 1011 | 1.00 | 1.00 | 1.00 | 0.99 | 0.98 | 0.90 | 0.68 | 0.33 | 0.10 | 0.03 |
| 1100 | 1.27 | 1.23 | 1.12 | 0.79 | 0.35 | 0.10 | 0.03 | 0.01 | 0.00 | 0.00 |
| 1101 | 1.28 | 1.27 | 1.22 | 1.06 | 0.68 | 0.26 | 0.07 | 0.02 | 0.00 | 0.00 |
| **1110** | 1.45 | 1.45 | 1.44 | 1.42 | 1.32 | 1.03 | 0.52 | 0.17 | 0.04 | 0.01 |
| **1111** | 1.25 | 1.25 | 1.25 | 1.25 | 1.24 | 1.20 | 1.05 | 0.69 | 0.28 | 0.08 |

**S2 Table.** **Simulated growth rates.** These are the growth rates generated from empirical parameters as discussed in the **Methods** section.
