# Supplementary material for: Adaptive Landscape by Environment Interactions Dictate Evolutionary Dynamics in Models of Drug Resistance
Source: PLoS Comput Biol. 2016 Jan 25;12(1):e1004710. doi: 10.1371/journal.pcbi.1004710 (PMC4726534; doi:10.1371/journal.pcbi.1004710)
Supplement: S3 Table — (DOCX) [file pcbi.1004710.s005.docx]

| Pathways as depicted in Figure 3 (main text) | |
| --- | --- |
| a | 0000-1000-1100-1110-1111 |
| b | 0000-1000-1100-1101-1111 |
| c | 0000-1000-1010-1110-1111 |
| d | 0000-1000-1010-1011-1111 |
| e | 0000-1000-1001-1101-1111 |
| f | 0000-1000-1001-1011-1111 |
| g | 0000-0100-1100-1110-1111 |
| h | 0000-0100-1100-1101-1111 |
| i | 0000-0100-0110-1110-1111 |
| j | 0000-0100-0110-0111-1111 |
| k | 0000-0100-0101-1101-1111 |
| l | 0000-0100-0101-0111-1111 |
| m | 0000-0010-1010-1110-1111 |
| n | 0000-0010-0110-1110-1111 |
| o | 0000-0010-0110-1110-1111 |
| p | 0000-0010-0110-0111-1111 |
| q | 0000-0010-0011-1011-1111 |
| r | 0000-0010-0011-0111-1111 |
| s | 0000-0001-1001-1101-1111 |
| t | 0000-0001-1001-1011-1111 |
| u | 0000-0001-0101-1101-1111 |
| v | 0000-0001-0101-0111-1111 |
| w | 0000-0001-0011-1011-1111 |
| x | 0000-0001-0011-0111-1111 |

**S3 Table**. **Discrete pathways with letter corresponding to the paths in Figure 3.**
