# Supplementary material for: Adaptive Landscape by Environment Interactions Dictate Evolutionary Dynamics in Models of Drug Resistance
Source: PLoS Comput Biol. 2016 Jan 25;12(1):e1004710. doi: 10.1371/journal.pcbi.1004710 (PMC4726534; doi:10.1371/journal.pcbi.1004710)
Supplement: S4 Table — All values for df num., denom. = 9, 70. (DOCX) [file pcbi.1004710.s006.docx]

**ANOVA**

Effect *F P*

**Pyrimethamine**
Absolute effect of 1*** X environment 0.34 0.96

Absolute effect of *1** X environment 0.80 0.62

Absolute effect of **1* X environment 4.57 0.00009

Absolute effect of ***1 X environment 0.71 0.70

Scaled effect of 1*** X environment 1.07 0.39

Scaled effect of *1** X environment 1.79 0.09

Scaled effect of **1* X environment 8.10 4.0 X 10^-8^

Scaled effect of ***1 X environment 0.11 0.99

**Cycloguanil**

Absolute effect of 1*** X environment 0.60 0.79

Absolute effect of *1** X environment 0.32 0.97

Absolute effect of **1* X environment 3.68 0.00081

Absolute effect of ***1 X environment 2.01 0.050

Scaled effect of 1*** X environment 1.11 0.37

Scaled effect of *1** X environment 0.87 0.56

Scaled effect of **1* X environment 1.98 0.054

Scaled effect of ***1 X environment 1.63 0.12

**S4 Table. ANOVA**: **Interaction between mutation effect and drug concentration for both pyrimethamine and cycloguanil.** All values for df _num., denom._ = 9, 70
