# Supplementary material for: Adaptive Landscape by Environment Interactions Dictate Evolutionary Dynamics in Models of Drug Resistance
Source: PLoS Comput Biol. 2016 Jan 25;12(1):e1004710. doi: 10.1371/journal.pcbi.1004710 (PMC4726534; doi:10.1371/journal.pcbi.1004710)
Supplement: S5 Table — This analysis directly investigates the ratio between experimental noise and the dispersion of fitness effects for all measurable alleles in the drugless environment (where fitness values are the highest for all alleles). The table indicates that for 14/15 alleles with measured fitness values, the standard error in the fitness measurements is smaller than the standard error of the fitness effects of mutations (ratio < 1.0), justifying the study of the landscape in terms of discrete alleles. (DOCX) [file pcbi.1004710.s007.docx]

| Allele | S.E. Growth Rates | S.E. Fitness Effects | Ratio |
| --- | --- | --- | --- |
| 0000 | 0.0535 | 0.694 | 0.0771 |
| 0001 | 0.0131 | 0.333 | 0.0393 |
| 0010 | 0.0195 | 0.342 | 0.0569 |
| 0011 | No growth | 0.061 | - |
| 0100 | 0.0287 | 0.030 | 0.9664 |
| 0101 | 0.0164 | 0.031 | 0.5271 |
| 0110 | 0.0268 | 0.056 | 0.4762 |
| 0111 | 0.0737 | 0.356 | 0.2069 |
| 1000 | 0.0349 | 0.066 | 0.5273 |
| 1001 | 0.0595 | 0.068 | 0.8779 |
| 1010 | 0.0336 | 0.096 | 0.3513 |
| 1011 | 0.0814 | 0.313 | 0.2603 |
| 1100 | 0.0509 | 0.071 | 0.7176 |
| 1101 | 0.0444 | 0.039 | 1.1242 |
| 1110 | 0.0159 | 0.032 | 0.4936 |
| 1111 | 0.0457 | 0.330 | 0.1386 |

**S5 Table. Ratio of experimental noise to measured fitness effects.** This analysis directly investigates the ratio between experimental noise and the dispersion of fitness effects for all measurable alleles in the drugless environment (where fitness values are the highest for all alleles). The table indicates that for 14/15 alleles with measured fitness values, the standard error in the fitness measurements is smaller than the standard error of the fitness effects of mutations (ratio < 1.0), justifying the study of the landscape in terms of discrete alleles.
