# Supplementary material for: Adaptive Landscape by Environment Interactions Dictate Evolutionary Dynamics in Models of Drug Resistance
Source: PLoS Comput Biol. 2016 Jan 25;12(1):e1004710. doi: 10.1371/journal.pcbi.1004710 (PMC4726534; doi:10.1371/journal.pcbi.1004710)
Supplement: S6 Table — Average times to fixation are displayed for major pathways, which demonstrates how the timescale of evolution changes as a function of environment. ANOVA results compare the fixation times for preferred pathways a-g. (DOCX) [file pcbi.1004710.s008.docx]

| **Preferred Pathways and frequency of runs that they occur** | | | |
| --- | --- | --- | --- |
| **No drug** | | **Fraction** | **Avg. Time to Fixation (S.D.)** |
| 1° pathway | 0000 (Never leaves ancestor) | 0.92^a^ | 0 |
| 2° pathway | 0000🡪0100🡪0110🡪1110 | 0.07 | - |
| **Pyrimethamine, low drug (1 uM)** | | **Fraction** | **-** |
| 1° pathway | 0000🡪0010🡪0110🡪1110 | .97^b^ | 368.4 (81.7) |
| 2° pathway | 0000🡪0100🡪0110🡪1110 | .02 | - |
| **Pyrimethamine, Intermediate drug (100 uM)** | | **Fraction** |  |
| 1° pathway | 0000🡪0010🡪0110🡪1110 | 0.96^c^ | 127.1 (40.8) |
| 2° pathway | 0000🡪0100🡪0110🡪1110 | 0.03 | - |
| **Pyrimethamine, High drug (10,000 uM)** | | **Fraction** | **-** |
| 1° pathway | 0000🡪0010🡪0110🡪1110🡪1111 | .97^d^ | 108.9 (34.2) |
| 2° pathway | 0000🡪0100🡪0110🡪1110 | .02 | - |
| **Cycloguanil, Low drug (1 uM)** | | **Fraction** |  |
| 1° pathway | 0000🡪0010🡪0110 | 0.96^e^ | 56.5 (32.4) |
| 2° pathway | 0000🡪0010🡪0110🡪0111 | 0.03 | - |
| **Cycloguanil, Intermediate drug (100 uM)** | | **Fraction** |  |
| 1° pathway | 0000🡪0010🡪0110🡪0111 | .99^f^ | 66.8 (29.1) |
| 2° pathway | - |  |  |
| **Cycloguanil, High drug (10,000 uM)** | | **Fraction** |  |
| 1° pathway | 0000🡪0010🡪0110🡪0111 | .99^g^ | 18.5 (7.1) |
| 2° pathway | - | - |  |
|  |  |  |  |
| **ANOVA** | |  |  |
| Fixation times for preferred pathways differ across environments (a-g) | | df _num., denom._ | 5, 564 |
|  |  | *F* | 763.36 |
|  |  | *P* | < 0.0000001 |

^a-g^Correspond to preferred pathways at each drug environment

**S6 Table.** **Summary of simulations of evolution across several drug concentrations.** Average times to fixation are displayed for major pathways, which demonstrates how the timescale of evolution changes as a function of environment. ANOVA results compare the fixation times for preferred pathways a-g.
