# Supplementary material for: Adaptive Landscape by Environment Interactions Dictate Evolutionary Dynamics in Models of Drug Resistance
Source: PLoS Comput Biol. 2016 Jan 25;12(1):e1004710. doi: 10.1371/journal.pcbi.1004710 (PMC4726534; doi:10.1371/journal.pcbi.1004710)
Supplement: S1 References — (DOCX) [file pcbi.1004710.s009.docx]

**S1 References.** This is a list of references that corresponds to findings from field studies of *P. falciparum* DHFR mutations with varying resistance to pyrimethamine.

In these studies, alleles highlighted in Table 1 and mentioned in the main text (see: **Discussion**) were isolated. Many correspond to alleles that are either fitness peaks at various environments or are “stepping stone” alleles in the evolution towards environment-specific peaks as observed in our study. Please note that this literature is vast, with many of the more resistant mutants found in hundreds of different settings, with new studies published quite often.

| **Allele** | **Supplementary References** |
| --- | --- |
| 0010 | [1,2] |
| 0110 | [3,4] |
| 1010 | [5,6] |
| 0111 | [7,8] |
| 1110 | [3,4] |
| 1111 | [9,10] |

1. Saito-Nakano Y, Tanabe K, Kamei K, Iwagami M, Komaki-Yasuda K, Kawazu S, et al. Genetic evidence for Plasmodium falciparum resistance to chloroquine and pyrimethamine in Indochina and the Western Pacific between 1984 and 1998. Am J Trop Med Hyg. 2008 Oct;79(4):613–9.

2. Saito-Nakano Y, Tanabe K, Mita T. Identification of pyrimethamine- and chloroquine-resistant Plasmodium falciparum in Africa between 1984 and 1998: genotyping of archive blood samples. Malar J. 2011;10:388.

3. Nwakanma DC, Duffy CW, Amambua-Ngwa A, Oriero EC, Bojang KA, Pinder M, et al. Changes in malaria parasite drug resistance in an endemic population over a 25-year period with resulting genomic evidence of selection. J Infect Dis. 2014 Apr 1;209(7):1126–35

4. Iriemenam NC, Shah M, Gatei W, van Eijk AM, Ayisi J, Kariuki S, et al. Temporal trends of sulphadoxine-pyrimethamine (SP) drug-resistance molecular markers in Plasmodium falciparum parasites from pregnant women in western Kenya. Malar J. 2012;11:134.

5. Tinto H, Ouédraogo JB, Zongo I, van Overmeir C, van Marck E, Guiguemdé TR, et al. Sulfadoxine-pyrimethamine efficacy and selection of Plasmodium falciparum DHFR mutations in Burkina Faso before its introduction as intermittent preventive treatment for pregnant women. Am J Trop Med Hyg. 2007 Apr;76(4):608–13.

6. Certain LK, Briceño M, Kiara SM, Nzila AM, Watkins WM, Sibley CH. Characteristics of Plasmodium falciparum dhfr haplotypes that confer pyrimethamine resistance, Kilifi, Kenya, 1987--2006. J Infect Dis. 2008 Jun 15;197(12):1743–51.

7. Heidari A, Dittrich S, Jelinek T, Kheirandish A, Banihashemi K, Keshavarz H. Genotypes and in vivo resistance of Plasmodium falciparum isolates in an endemic region of Iran. Parasitol Res. 2007 Feb;100(3):589–92.

8. Lau TY, Sylvi M, William T. Mutational analysis of Plasmodium falciparum dihydrofolate reductase and dihydropteroate synthase genes in the interior division of Sabah, Malaysia. Malar J. 2013 Dec 10;12(1):445.

9. Huang F, Tang L, Yang H, Zhou S, Liu H, Li J, et al. Molecular epidemiology of drug resistance markers of Plasmodium falciparum in Yunnan Province, China. Malar J. 2012;11:243.

10. Ahmed A, Das MK, Dev V, Saifi MA, Wajihullah null, Sharma YD. Quadruple mutations in dihydrofolate reductase of Plasmodium falciparum isolates from Car Nicobar Island, India. Antimicrob Agents Chemother. 2006 Apr;50(4):1546–9.
